# Scale‐space image analysis for mapping spotted knapweed in grassland ecosystems

**DOI:** 10.1002/aps3.70086

**Published:** 2026-10-14

**Authors:** Shohreh Sahebi, David J. Hill

**Affiliations:** ^1^ Master of Science in Environmental Science Program Thompson Rivers University Kamloops British Columbia Canada; ^2^ Department of Environment, Culture, and Society Thompson Rivers University Kamloops British Columbia Canada

**Keywords:** *Centaurea stoebe*, Gaussian pyramid, invasive species, random forest, scale space, unpiloted aerial vehicle (UAV)

## Abstract

**Premise:**

This study investigates the value of scale‐space representations of spatial features for the identification and mapping of invasive plants using high‐resolution multispectral imagery. Scale‐space representations combine the two‐dimensional spatial domain with a third dimension representing spatial scale, such that features can be represented across both space and image spatial resolution.

**Methods:**

Gaussian pyramids were constructed to create representations of the features across five image resolution levels (between 3 and 46 cm) within the scale space. A case study was employed to evaluate the importance of features across the scale space for the identification and mapping of spotted knapweed in a grassland ecosystem using multispectral imagery acquired using a remotely piloted aircraft system (RPAS). Classification was performed using a random forest classifier that was constructed using a process that included feature and hyperparameter optimization.

**Results:**

The results of this case study show that classifiers constructed with only high spatial resolution features were associated with better classification performance than those constructed with only lower spatial resolution features; however, the best classification is achieved by leveraging features from throughout the feature scale spaces.

**Discussion:**

These findings suggest that optimization of the spatial scale at which features are computed can substantially improve remote sensing image analysis and potentially inform RPAS flight planning to improve mission efficiency.

Grasslands constitute some of the largest ecosystems worldwide (Suttie et al., 2005) and provide irreplaceable ecosystem services (O'Mara, 2012). However, human pressures on grasslands inadvertently promote the spread of invasive plants, which displace native species, inhibit the functioning of these ecosystems, and ultimately endanger their survival (Samson and Knopf, 1994; Foster et al., 2020; Gaskin et al., 2021). In the grasslands of the southern interior of British Columbia (BC), Canada, spotted knapweed (*Centaurea stoebe* L.) is identified as a particularly virulent invasive plant and prioritized for containment and control (Inter‐Ministry Invasive Species Working Group, 2025). Spotted knapweed is a perennial that forms a rosette and a deep taproot from which multiple flowering stems emerge. By producing many seeds and releasing allelopathic compounds into the soil that suppress other vegetation, spotted knapweed is able to spread rapidly across disturbed sites and outcompete native vegetation within BC grasslands. Spotted knapweed leaves can reach lengths of 15 cm at the rosette and decrease in size moving towards the stem tip. Purple thistle‐like flowers and black‐tipped bracts make spotted knapweed easily visible when flowering, which occurs in August through September, depending on altitude. However, because spotted knapweed is a prolific seed producer and seeds are shed as soon as they reach maturity, it is important to detect and map spotted knapweed invasions before flowering so control measures can be conducted before seed formation (British Columbia Ministry of Agriculture, Food, and Fisheries, 2002).

A key aspect of managing an invasive plant, such as spotted knapweed, is detection and mapping of the invasion at the field scale. Historically, field‐based visual inspections and plant species inventories have served this purpose, but these methods are time consuming and impractical for large areas (Bradley, 2014; Hill et al., 2017). To address these limitations, remote sensing technology, known for its ability to collect data over vast spatial extents, has been extensively explored for detecting and mapping vegetative species invasions (Huang and Asner, 2009; Bradley, 2014; Baron and Hill, 2020; Gholizadeh et al., 2022). The primary challenge in vegetation mapping using remote sensing is differentiating target plants from background vegetation. This challenge is even more pronounced in grasslands due to the small size and sparse canopy of grassland plant species, as well as the variety of different plants growing in the community (Malanson and Walsh, 2013). Effective detection often necessitates that the invasive plants form relatively homogeneous patches with dense canopies, leaves, or other structures that are reasonably uniform and larger than the spatial resolution of the remote sensing imagery (He et al., 2015). Thus, image spatial resolution is a critical variable that must be selected based on the intended target species of the analysis (Underwood et al., 2007).

With resolutions on the order of meters, imagery from satellite‐based remote sensors is ill‐suited for mapping invasive plants in grassland ecosystems, particularly in the early stages of invasion (Malanson and Walsh, 2013). Due to the high spatial resolution of imagery acquired by remotely piloted aircraft systems (RPASs), which can achieve millimeter resolution sufficient to detect herbaceous plants like spotted knapweed, there has been increasing interest in using these data for monitoring and mapping invasive plants (Dvořák et al., 2015; Hill et al., 2017; Lehmann et al., 2017; Baron et al., 2018; Baron and Hill, 2020; Mallmann et al., 2020). Another significant benefit of RPASs is the ease and flexibility in deploying these systems, which enables researchers and land managers to conduct imaging missions with high frequency, increasing the temporal resolution of the collected data (Klosterman and Richardson, 2017; Klosterman et al., 2018; Hill et al., 2019).

Imagery acquired by RPASs presents an opportunity to apply remote sensing methods to monitoring and mapping an increasing variety of invasive species in grassland ecosystems. These data have already shown promise for detecting and measuring target species, including spotted knapweed, within treatment areas (Hill et al., 2017; Tamouridou et al., 2017; Martin et al., 2018), particularly when dealing with very small and/or very sparse canopies (Malanson and Walsh, 2013; Gholizadeh et al., 2022). However, the identification of a target species against a heterogeneous background of other vegetation and bare soil remains challenging.

To address this issue, Baron and Hill (2020) proposed a method they called metapixel‐based image classification. Metapixel‐based image classification leverages the principles of object‐based image analysis to identify the relative abundance of an invasive plant in a grassland ecosystem. In this method, chessboard segmentation is used to divide the image into non‐overlapping squares, termed metapixels. These metapixels are larger than the image resolution and correspond to the size (1 m^2^) of quadrats used by range managers in the field to determine the relative abundance of the target plant species. Baron and Hill (2020) used these metapixels to compute aggregate features that aided image classification. These features constitute second‐order statistics (i.e., a statistic that describes a relationship between pairs of observations) computed from the gray‐level co‐occurrence matrices (GLCMs) of each spectral band. The GLCM of an image quantifies spatial relationships within an image or subimage by counting the number of times specific values are found separated by a distance *d* in a particular direction α. These GLCM‐based statistics quantify image texture (Haralick et al., 1973; Conners and Harlow, 1980; Tahir et al., 2003; Kekre et al., 2010) and have been shown to substantially increase the prediction of the relative abundance of spotted knapweed in each metapixel (Baron and Hill, 2020).

GLCM‐based texture features are dependent on the spatial resolution of the image from which they are derived. This is because the spatial structure of objects captured within a remote sensing image is limited by the spatial resolution of the image itself. Structures with spatial scales smaller than the spatial resolution are filtered out of the image, so that the image‐derived features are focused on structures larger than the spatial resolution of the image. Thus, the spatial resolution of the image data functions as a low‐pass spatial filter that removes shorter spatial patterns that lead to high‐frequency spatial variation in the image. Both small‐ and large‐scale patterns can be identified within high‐resolution imagery. While patterns with a characteristic length scale on the order of the image pixel size can be identified using one pixel‐width as the GLCM separation distance, patterns that have a length scale larger than the pixel size will be more difficult to calculate, requiring GLCMs with larger separation distances. Additionally, unless the small‐scale features identified are relevant to the classification task, they may simply constitute noise that must be filtered out by the classifier. On the other hand, lower image resolutions filter out small‐scale patterns with length scales smaller than the pixel size. If these small‐scale features are irrelevant to the classification task, this filtering performed by the image acquisition process will increase the signal‐to‐noise ratio of the derived image features. The larger pixel size of the lower‐resolution image also makes it easier to quantify patterns with length scales on the order of the larger pixel size using a GLCM. However, this comes at the expense of obscuring small‐scale patterns that are important to the analysis. Thus, the desired image resolution is tied to the characteristic length scales of the features that are needed to drive the classification model.

To address this issue, scale‐space theory advocates for simultaneous representation of a scene across all spatial scales, offering a structured methodology to represent a scene as a continuum of smoothed (i.e., blurred) versions across all spatial scales (Witkin, 1983; Koenderink, 1984; Florack et al., 1992; Romeny, 2008; Lindeberg, 1990, 1995, 2009). Just as a digital image is a discrete space representation of the continuous scene, the continuum of blurred scenes that comprise a scene's scale space can be represented as a finite set of images spanning the range of spatial scales from the original image resolution to a homogeneous field represented as a single pixel that encompasses the entire scene. Creation of a scale‐space representation of an image is accomplished by convoluting the image with kernels of increasing size to produce a series of increasingly smoothed versions of the original image (Kuijper et al., 2003).

By applying scale‐space theory, this research seeks to establish whether there is a relationship between spatial resolution and image classification accuracy that can help determine whether (1) the optimal spatial resolution for a given classification task can be defined and identified, based on classifier accuracy, and (2) providing image features to a classifier at multiple spatial resolutions will improve or hinder classification accuracy beyond what can be achieved with single‐resolution data. These questions will be addressed in the context of mapping spotted knapweed using metapixel image analysis (Baron and Hill, 2020).

## METHODS

### Data acquisition and image processing

The field sites investigated are located within the Laurie Guichon Memorial Grasslands Interpretive Site (LGMGIS) south of Merritt, BC, Canada. This 100‐ha site is situated in Canada's Western Cordillera physiographic region (Bone, 2003) and is classified as representing the Thompson Very Dry Hot Interior Douglas‐fir Variant of BC's Interior Douglas‐fir biogeoclimactic zone (IDFxh2; Grasslands Conservation Council of British Columbia, 2017). Three field sites (Figure 1) were selected within the LGMGIS. Each field site covered an approximate area of 1 ha and exhibited a gentle slope with a southern aspect.

**Figure 1:**
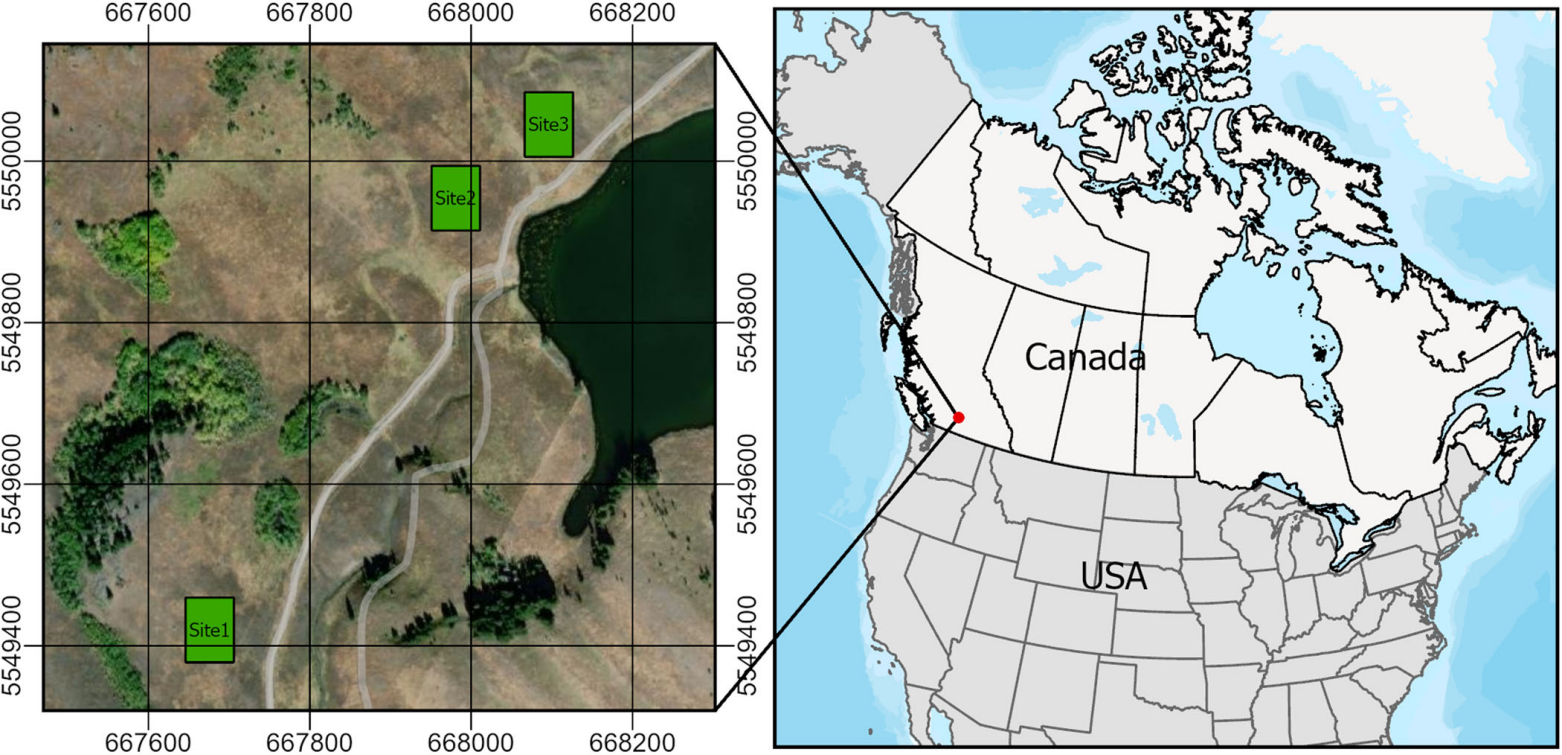
Locations of the three field sites in Laurie Guichon Memorial Grasslands Interpretive Site (LGMGIS). Basemap imagery is courtesy of Esri Canada (Toronto, Ontario, Canada) and Maxar Technologies (Westminster, Colorado, USA). The coordinate system is WGS UTM Zone 10N.

Ground reference data consisting of plant species inventories were collected within 93 randomly selected 1‐m^2^ quadrats (31 per site) on 12 July 2018. These inventories were created by direct inspection of the quadrats to determine the species present and the approximate percent canopy cover of each plant species within the quadrat boundaries. The quadrats were aligned with the cardinal (north/south and east/west) directions and marked with 70‐cm segments of rebar that were driven into the ground to mark the southwest corner of each quadrat. Six of the markers at each site functioned as ground control points (GCPs) for georeferencing the image data; GCPs were fitted with a 12.7‐cm diameter, high‐visibility safety‐orange cap. The location of the quadrats within field site 1 and an image of a typical quadrat acquired on 4 July 2018 are illustrated in Figure 2.

**Figure 2:**
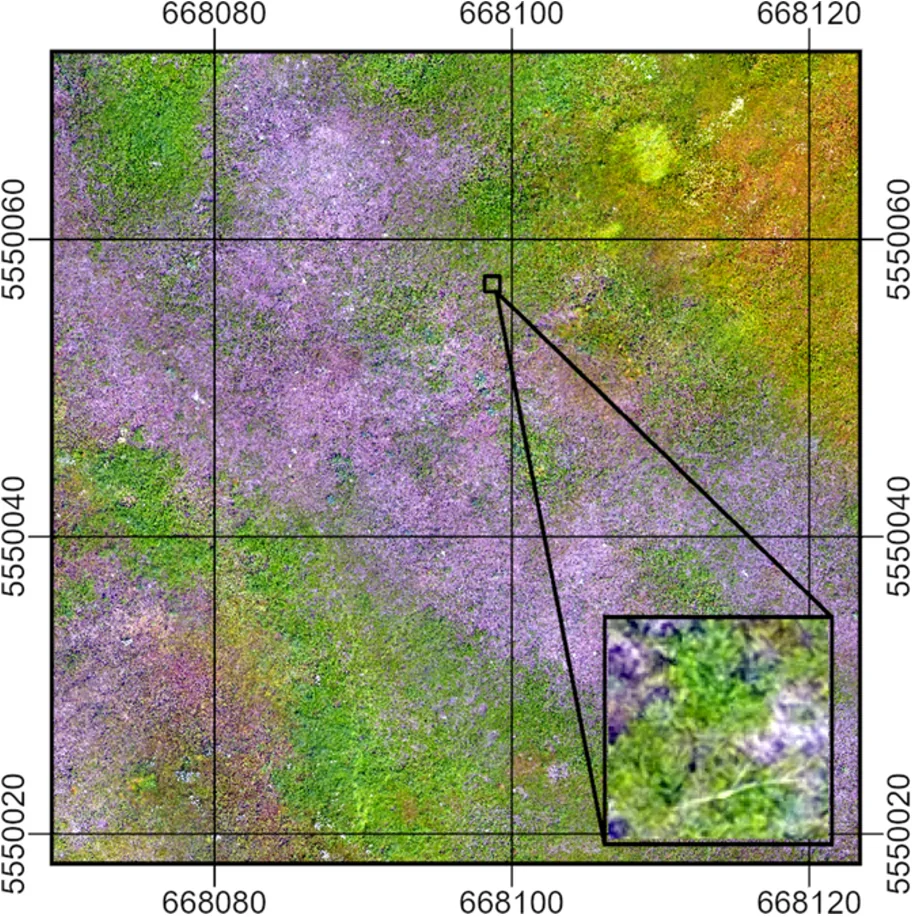
Locations of field quadrats at field site 3 showing an overlaid close‐up view of a standard quadrat recorded with a digital camera. The coordinate system is WGS UTM Zone 10N.

RPAS multispectral imagery was acquired at all three field sites using a Parrot Sequoia multispectral imager (Parrot, Paris, France) attached to a Phantom 4 quadcopter (Dà‐Jiāng Innovations Science and Technology Co. [DJI], Shenzhen, China). Imaging flights took place between 11:00 hours and 13:00 hours on 4, 12, and 19 July 2018. However, data were not acquired from site 1 on 12 July or from site 3 on 19 July, because the WiFi link between the RPAS and the iPad integrated into the RPAS control system could not be established on these days. Flight parameters are provided in Appendix S1 (see Supporting Information with this article). These flights produced image data covering 217 quadrats (31 per site) measured at all three sites on 4 July, at sites 2 and 3 on 12 July, and at sites 1 and 3 on 19 July.

The RPAS images were processed and combined with calibration data from a ground reflectance target to create orthomosaics of the spectral reflectance in the green, red, red‐edge, and near‐infrared (NIR) bands. These orthomosaics were then georeferenced using the six GCPs per field site and had a ground resolved distance (GRD) of 2.9 cm. All image processing was performed using the Pix4Dmapper software (educational license; Pix4D, Prilly, Switzerland). Further details on data collection and preprocessing can be found in Baron and Hill (2020). These data are openly accessible for download via the Open Science Framework (OSF) (see Data Availability Statement).

### Metapixel‐based image classification

Metapixel‐based classification (Baron and Hill, 2020) requires segmenting the multispectral image data into non‐overlapping squares called metapixels. To avoid representativeness issues that would result from changing the spatial support of the relative abundance calculation between the field‐ and image‐based methods, the size of the metapixels is selected to be consistent with the size of the quadrats used for the field data collection (i.e., 1 m^2^). Each metapixel was then described using features derived from the image pixels within the metapixel.

A random forest classifier was selected to perform image classification because this type of classifier has been used extensively in remote sensing applications (Marceau et al., 1990; Breiman, 2001) and has been successful in the identification of a wide variety of invasive plant species from remote sensing imagery (Michez et al., 2016; Baron et al., 2018; Martin et al., 2018). The random forest classifier was trained to predict the relative abundance of spotted knapweed within a metapixel based on the input features. The random forest implementation in the Scikit‐learn 0.20.1 machine learning library (Pedregosa et al., 2011) was used. Optimization of the random forest hyperparameters, listed in Appendix S2, was conducted using a grid search guided by the mean cross‐validation scores of the classifiers trained using each hyperparameter combination, and the overall performance of the optimized classifier was quantified by testing the tuned classifiers on a validation dataset that had been held out of the training process. The maximum number of features used to grow individual trees within the forest was set using the square‐root rule, as this was found to produce good performance (Probst et al., 2019).

### Image scale space and Gaussian pyramids

Objects and landscape features present in the environment possess multi‐scale spatial characteristics and thus will appear differently depending on the scale at which they are viewed (Turner et al., 2001; Zhang and Li, 2020). However, without prior knowledge, determining relevant spatial scales for analysis is challenging if not impossible. Instead, scale‐space theory posits that a digital image constitutes one member of a family of images that exist along a continuum that can be represented by a single value related to the pixel size (Lindeberg, 1994; Florack, 1997; Sporring et al., 2013). Thus, any specific image of a scene is one of the many possible images of the scene within the image's scale space.

A Gaussian pyramid (GP) is a scale‐space representation of an image that is created by recursively convoluting an image with a Gaussian kernel to produce a sequence of increasingly blurred images. The family of Gaussian kernels has been widely adopted for image smoothing because they have several beneficial properties for image smoothing, namely, linearity, separability, causality, and the semi‐group property (Witkin, 1983; Koenderink, 1984; Florack et al., 1992; Lindeberg, 1995; Sporring et al., 2013).

The two‐dimensional Gaussian function used to define the kernel is:

(1) $$g\left(x,y,\sigma ,\mu_{x},\mu_{y}\right)=\;\frac{1}{2\pi \sigma^{2}}\;{\text{exp}}^{\left(-\frac{\;{\left(x-\mu_{x}\right)}^{2}+\;{\left(y-\mu_{y}\right)}^{2}}{{2\sigma }^{2}}\right)}$$

where x and y are the coordinates of the location of interest relative to the center of the Gaussian function, respectively; σ is the standard deviation of the Gaussian function; and $\mu_{x}$ and $\mu_{y}$ are the *x*‐ and *y*‐coordinate of the center of the Gaussian function, respectively. To create a GP, the kernel is defined to be symmetric in the *x* and *y* dimensions and centered on each pixel center. Thus, the kernel has only one parameter σ which defines the degree of blurring that the kernel produces (Chaudhuri and Marron, 2000; Binaghi et al., 2003). The separability property of Gaussian kernels permits the two‐dimensional Gaussian kernel to be decomposed into two orthogonal, one‐dimensional kernels. This significantly reduces the computational complexity of applying a Gaussian kernel, because it allows the smoothing of an image to be accomplished through two separate one‐dimensional smoothing steps, each applied to one dimension of the image (Mikolajczyk, 2002).

The separability property of Gaussian kernels also permits GPs to be constructed through a recursive algorithm (Deriche, 1993). During each recursion of this algorithm, an image, referred to as the level L(l) representation of the image pyramid, is convoluted with a Gaussian kernel, centered on each image pixel. Post‐blurring, the image is downsampled by merging each 2 × 2 block of image pixels and assigning them the value of the convoluted pixels producing the level L(l+1) image. Because of the downsampling, the level L(l+1) has four times fewer pixels than the level L(l) image and thus the spatial extent of each level L(l+1) pixel is four times larger than the level L(l) pixels (Li et al., 2018). This process is applied recursively starting with the original image (defined as the level L0 image) until the entire scene is represented by a single pixel. Stacking the increasingly coarse level images results in a pyramid structure (see Figure 3). This layered representation reduces high‐frequency variation (e.g., noise) in the image as pyramid levels increase by enhancing its smoothness (Li et al., 2018).

**Figure 3:**
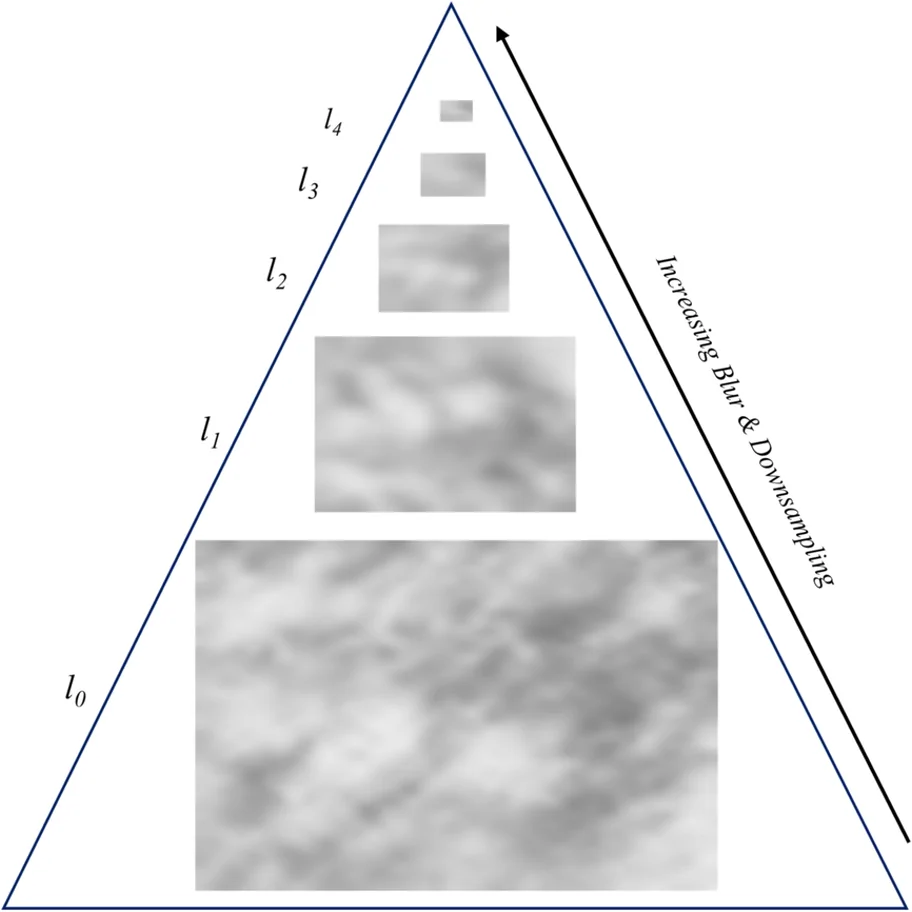
Illustration of a Gaussian pyramid (GP), with five levels spanning from the original image at level L0 to the fifth level (L4). Ascending from the base of the pyramid to the apex, the image dimensions in number of pixels decrease from 34 × 34 to 2 × 2, with each successive image having half as many pixels as the one below it in the pyramid. As the number of pixels in an image decreases, the spatial extent per pixel increases from 2.9 × 2.9 cm to 46 × 46 cm to maintain the spatial extent of the overall image. This reduction in spatial resolution from the base of the pyramid to the apex is visible as increasing blurring of the image.

Because digital images discretize space into pixels, to create a GP of digital images, the Gaussian kernel must also be discretized. Following Bradski (2000), this work employs a discrete two‐dimensional Gaussian kernel that spans a 5 × 5‐pixel neighborhood:

(2) $$1/256\left[\begin{array}{ccccc} 1 & 4 & 6 & 4 & 1\\ 4 & 16 & 24 & 16 & 4\\ 6 & 24 & 36 & 24 & 6\\ 4 & 16 & 24 & 16 & 4\\ 1 & 4 & 6 & 4 & 1 \end{array}\right]$$

The factor 1/256 normalizes the kernel, ensuring that the sum of all weights is 1. Convolving this discrete kernel with a 5 × 5‐pixel neighborhood in an image results in the computation of a weighted average of the pixel values, where the weight is defined by the kernel. Image GPs were computed using tools available in the Scikit‐Image 0.14.1 image processing library (Van der Walt et al., 2014).

### Feature creation

Ground reference data provided the on‐the‐ground conditions within each quadrat that the random forest model was constructed to predict. Given the variability in spotted knapweed density across the surveyed quadrats and the relatively small number of quadrats surveyed, percent cover of spotted knapweed within each quadrat was categorized into classes. Quadrats in which spotted knapweed was not present or only present in trace amounts were classified as “Low,” quadrats in which spotted knapweed did not exceed 25% cover were classified as “Moderate,” and quadrats in which spotted knapweed exceeded 25% cover were classified as “High.” These breakpoints roughly align with the 33rd and 66th percentiles of the relative abundance data, providing a comprehensible threshold for dividing the data into approximate thirds. The complete dataset consisted of 217 measured quadrats, 63 classified as Low, 87 classified as Moderate, and 67 classified as High.

From the green (G), red (R), red‐edge (RE), and near‐infrared (NIR) band images, three vegetation indices, adaptations of the normalized difference vegetation index (NDVI), were calculated:

(3) $$\text{NDVI}\;=\frac{\rho_{\mathrm{NIR}}-\rho_{R}}{\rho_{\mathrm{NIR}}+\rho_{R}}$$

(4) $$\text{gNDVI}\;=\frac{\rho_{\mathrm{NIR}}-\rho_{G}}{\rho_{\mathrm{NIR}}+\rho_{G}}$$

(5) $$\text{reNDVI}\;=\frac{\rho_{\mathrm{NIR}}-\rho_{\mathrm{RE}}}{\rho_{\mathrm{NIR}}+\rho_{\mathrm{RE}}}$$

where $\rho_{G}$, $\rho_{R}$, $\rho_{\mathrm{RE}}$, and $\rho_{\mathrm{NIR}}$ are the green, red, RE, and NIR spectral reflectance values. Rasters of these vegetation indices were created with the same spatial resolution (2.9 cm) as the spectral data rasters.

The scale space of each spectral band and multispectral index raster was then represented using a GP with five levels (L0–L4). The pixel ground sampling distance (GSD) and number of pixels representing a single 1‐m^2^ quadrat at each level in the GP are listed in Table 1. The mean, standard deviation, and five second‐order spatial statistics were used to summarize the information in each metapixel to create descriptive features to use for classification. The five second‐order spatial statistics are based on the GLCM of an image. A separation distance of one pixel was used to calculate the GLCM used to derive textural features. This separation distance defined a kernel over which the GLCM was computed. The directional relationship between pixels was defined by four different directions across an image: horizontal, vertical, left diagonal, and right diagonal. Because these angular relationships are reciprocal, the resultant GLCM was symmetric. This means patterns observed in one direction (e.g., up) were similar to its counterpart (e.g., down), forming a symmetrical relationship, whether horizontal, vertical, or diagonal. Specifically, the angular second moment (ASM), correlation (COR), entropy (ENT), sum entropy (SENT), and difference entropy (DENT) statistics were used. These features were selected because they are invariant to gray‐tone transformations (Haralick et al., 1973; Gonzalez and Woods, 2008) and thus were expected to be less sensitive to changes in illumination (e.g., shadows) and calibration errors.

**Table 1:** Ground sampling distance (GSD), number of pixels, and dimensions of each 1‐m^2^ quadrat at each level of the raster image Gaussian pyramids (GPs).

GP level	Pixel GSD (cm)	Number of pixels (Dimensions per 1‐m^2^ quadrat)
L0	2.9	1156 (34 × 34)
L1	5.8	289 (17 × 17)
L2	12	64 (8 × 8)
L3	23	16 (4 × 4)
L4	46	4 (2 × 2)

The GLCM‐based statistics were calculated using tools in the Scikit‐Image 0.14.1 image processing library (Van der Walt et al., 2014). Equations for these statistics are reported in Appendix S3. These statistics were computed for each directional GLCM, creating four directional variants (vertical, horizontal, diagonal‐up, diagonal‐down) of the statistic. These directional variants were summarized by the mean and range statistics to create omnidirectional versions of the GLCM‐based spatial statistics. Mean and range statistics were selected because they are invariant under rotation (Haralick et al., 1973) of the metapixel and thus were expected to generalize across a landscape where geographic features are not expected to be co‐aligned. Therefore, at each level in the GP, 12 statistics (see Appendix S4) were computed to summarize each of the four spectral reflectance and three vegetation index values of the pixels within each quadrat, resulting in a set of 84 features per quadrat. When computed across the five spatial scales represented by the GPs, each quadrat was represented by a total of 420 scale‐space features. To explore the scale‐space representation of the features, five single‐scale feature sets were created using data from each level in the GPs, and one scale‐space feature set was created that combined features from all the levels in the GPs. Thus, the single‐scale feature sets contained 84 features per quadrat, while the scale‐space feature set contained 420 features that comprise the 84 single‐scale features at each of the five levels of the GP.

### Ensemble feature selection

Studies have shown that reducing the dimensionality of the input data via feature selection can increase classification performance (Chang et al., 2019; Baron and Hill, 2020; Misra and Yadav, 2020). A feature selection method was performed that employed ensemble estimation to address the stochasticity inherent in random forest training and cross‐validation procedures. This feature selection method sampled outcomes of recursive feature elimination (RFE) to identify the optimal number of features and the identity of these features. Recursive feature elimination generates a feature subset by systematically removing the least important feature from the set until the desired set size is reached (Svetnik et al., 2004; Ustebay et al., 2018). Feature importance was estimated via the out‐of‐bag error computed when constructing a random forest classifier using the input data. Out‐of‐bag error is an estimate of prediction error for ensemble methods (e.g., random forests) that is computed by predicting each training observation using only models that were trained using bootstrap samples of the training data that excluded that example. In RFE with cross‐validation (RFECV; Misra and Yadav, 2020), the data are divided into folds, RFE is performed on each fold, and the cross‐validation scores of the resulting classifiers are averaged.

Feature selection begins by repeating RFECV to construct many realizations of the best feature sets of all sizes (*n*_*x*_) from 1 to *n*_*f*_ features, where *n*_*f*_ is the maximum number of features available. For each feature set size (*n*_*x*_), RFECV was repeated 70 times, five‐fold cross‐validation was conducted, and these folds were shuffled between each iteration of RFECV. Upon completion of the repeated RFECV process, the distribution of mean cross‐validation scores for each potential feature set size was analyzed according to two criteria defining optimality of a feature set. Criterion 1 (c_1_) seeks to find an optimal set of size *n*_*opt*_ that is significantly more likely to produce a better classification than a smaller set (*n*_*x*_ *< n*_*x*_) by minimizing the number of smaller feature sets where the 75th percentile of the cross‐validation scores overlaps with the 25th percentile of the cross‐validation scores of the optimal set. Criterion 2 (c_2_) seeks to find an optimal set of size *n*_*opt*_ that is not significantly likely to produce a worse classification than a larger set (*n*_*x*_ > *n*_*opt*_) by minimizing the number of larger feature sets where the 25th percentile of the cross‐validation scores overlaps with the 75th percentile of the optimal set. These criteria can be represented as:

(6) $$x\in \left\{1,2,\ldots ,n\right\}argmin\_x:c_{1\;}\left[x\right]c_{1\;}\left[x\right]=\text{count}\left(q_{75}\left[i\right]>q_{25}\left[x\right]\right)\;\forall i\in \left\{1,2,\ldots ,(x-1)\right\}$$

(7) $$\mathrm{argmin}\_x:c_{c\;}\left[x\right]c_{2}\left[x\right]=\text{count}\left(q_{25}\left[i\right]>q_{75}\left[x\right]\right)\forall j\in \left\{(x+1),(x+2),\ldots ,n\right\}$$

where *q*_25_ and *q*_75_ represent the 25th and 75th percentile of the distribution of cross‐validation scores associated with a specific number of features in the random forest model, and count represents a function to count the number of instances where the inequality is true. Equations 6 and 7 constitute the objectives of a bivariate optimization for finding the optimal feature size. As is common in multi‐objective optimization, these two objectives conflict, meaning that a single best solution does not exist. Instead, there are several solutions to the optimization problem that represent different trade‐offs between minimizing c_1_ and minimizing c_2_. To evaluate these solutions, a Pareto efficiency approach was employed, which identifies the full set of optimal trade‐off solutions (i.e., all of the feature set sizes for which criteria c_1_ cannot be improved without negatively impacting criteria c_2_, and vice versa). These feature set sizes constitute the set of Pareto‐optimal solutions to the optimization problem that are neither better nor worse than each other according to the principle of Pareto efficiency.

To select a single solution to the problem to be used as the optimal feature set size (*n*_*opt*_), these Pareto‐optimal solutions were compared and the one that best balanced the competing objectives was selected. To do this, the Pareto‐optimal solutions were plotted in the c_1_ and c_2_ dimensions and the solution for *n*_*opt*_ was selected. When selecting *n*_*opt*_, preference was given to minimizing c_2_ at the expense of c_1_, because this ensured that there are few or no larger set sizes that are clearly superior to the selected optimal set size. In the case that more than one set met the selected c_1_ and c_2_ values, the set with the narrowest interquartile range (IQR) of cross‐validation scores was selected, because this narrower range of variability suggests that the training process produces a more consistently accurate classifier. Applying these decision rules to the set of Pareto‐optimal solutions identified the number of features, out of the full set, that are necessary to produce the “best” possible classifier based on the training data.

Once the optimal feature set size (*n*_*opt*_) was selected, the 70 feature sets of this size created by repeating RFECV were analyzed to identify the members of the optimal feature set. The membership of the optimal feature set was defined to be the *n*_*opt*_ features that were selected by the majority of these repeated feature optimization runs. Thus, the optimal feature set was identified by majority voting on the inclusion of individual features by each member of the ensemble of RFECV results.

## RESULTS

### Training and validation datasets

Following the work of Baron and Hill (2020), the data were split into two distinct subsets: a training set and a validation set. The training set comprised data from site 1 on 4 and 19 July, site 2 on 4 and 12 July, and site 3 on 12 and 19 July. To enhance the performance of the classifiers, undersampling was used to construct a balanced training set (Zhu et al., 2017). The resulting training set consisted of 150 samples in total: 50 in the High category, 50 in the Moderate category, and 50 in the Low category.

The validation set consisted solely of data collected on 4 July 2018 from the 31 quadrats in site 3. The class membership (i.e., High/Moderate/Low relative abundance of spotted knapweed) of these 31 examples is highly imbalanced, with 17 in the High category, 13 in the Moderate category, and 1 in the Low category. This imbalance was the result of selecting the quadrat locations at random throughout the site in the early spring, before vegetation had emerged. Despite this imbalance, these data were used for validation in order to be consistent with the work of Baron and Hill (2020), which provided a baseline model for classifying these data. Site 3 as imaged by the digital camera onboard the RPAS on this date is illustrated in Appendix S5.

### Feature selection

A random forest classifier composed of 500 trees was implemented to perform the ensemble RFECV‐based feature selection method developed for this study. The Pareto‐optimal solutions for the size of the GP level L0 features are illustrated in Figure 4. In this case, the solution where there were three feature sets that met criterion c_1_ and no feature sets that met criterion c_2_ was selected. This solution corresponds to a feature set size of six features. The distribution of cross‐validation scores for sets of L0 features shows that random forest models produced with six features selected by RFECV were highly likely (as indicated by the IQR) to be better than models produced with fewer features and no worse than models produced with more features (see Figure 5). The repeated RFECV curves for the other single‐scale feature sets and the scale‐space feature sets shared the same general pattern (see Figures 4 and 5). The feature set sizes and features selected for each single‐scale feature set and the multi‐scale feature set, along with the values of c_1_, c_2_, and the mean cross‐validation score, are shown in Table 2.

**Figure 4:**
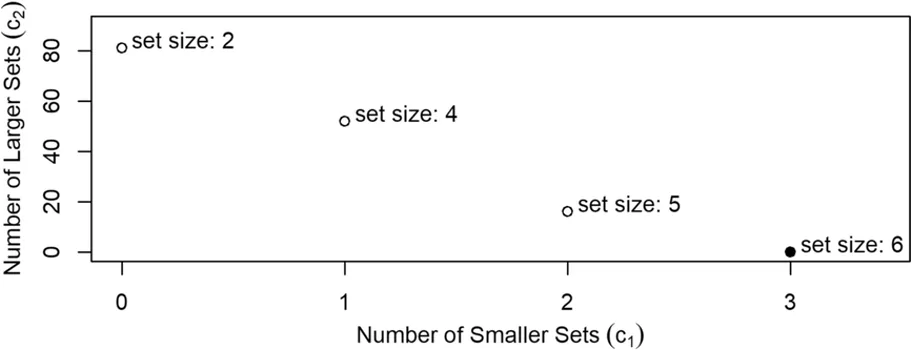
Pareto‐optimal solutions for the size of the GP L0 feature set produced using the ensemble method. The *x*‐ and *y*‐axes represent the two measures (c_1_ and c_2_), defined by Equations 6 and 7, that are minimized to find the optimal feature set size. For a particular feature set size, optimality measures c_1_ and c_2_ indicate the number of smaller and larger feature set sizes with similar performance, respectively. Thus, each point on this figure represents the best trade‐off that can be achieved between these two optimality measures, such that a further decrease in one measure would require an increase in the other. The point shaded in black indicates the solution for the feature set size selected as optimal.

**Figure 5:**
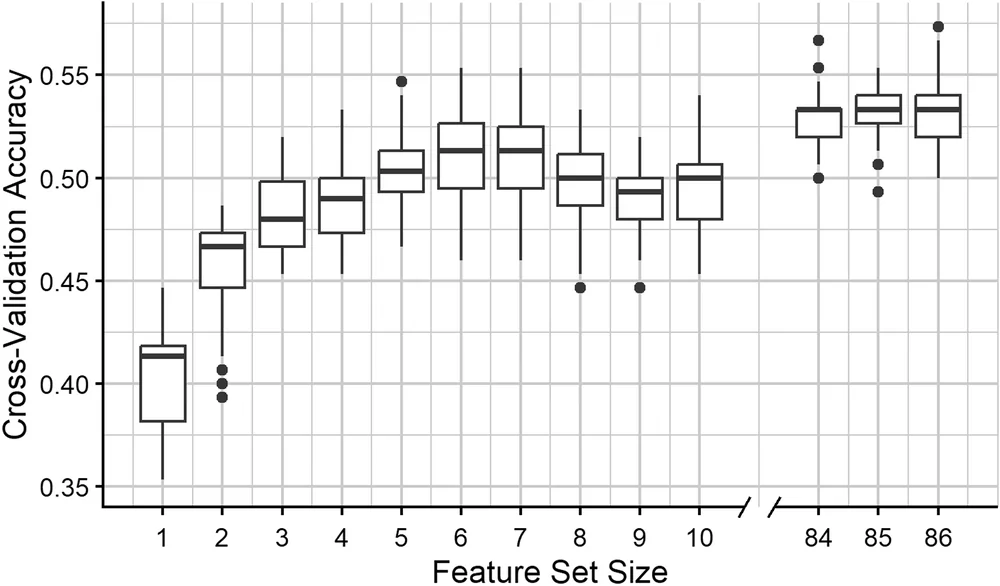
Distribution of cross‐validation scores (illustrated as boxplots) produced by the best Gaussian pyramid L0 feature sets of size 1–10 and 84–86 identified by 70 repetitions of recursive feature elimination with cross‐validation (RFECV). The *x*‐axis represents the feature set size, and the *y*‐axis represents the cross‐validation accuracy. The optimal feature set size, identified for the L0 features using the criteria listed in Equations 6 and 7, is 6.

**Table 2:** Optimal feature set size determined using ensemble feature selection, the values of the c_1_ and c_2_ optimality measures, and the mean cross‐validation score achieved over 70 iterations of recursive feature elimination with cross‐validation (RFECV).^a^

Level	Optimal number of features	c_1_	c_2_	Mean cross‐validation score
L0	6	3	0	0.513
L1	9	2	0	0.540
L2	3	0	0	0.500
L3	1	0	0	0.487
L4	1	0	0	0.520
Scale space	89	28	0	0.630

^a^Optimality measure c_1_ seeks to find an optimal set of features that is significantly more likely to produce a better classification than any smaller set, while measure c_2_ seeks to find an optimal set of features that is significantly more likely to produce a better classification than any larger set. The mean cross‐validation score represents the expected overall accuracy of a classifier trained using a set of features containing *n*_*opt*_ features, where *n*_*opt*_ is the number of optimal features listed in the second column of the table.

Identifying the optimal feature set size *n*_*opt*_ was the first step towards determining the identities of the features that constitute the optimal feature set of size *n*_*opt*_, which were needed to train the classifier. Once the size of the optimal feature set was identified for each feature set, the features that constitute the optimal feature set for each dataset were selected by identifying the *n*_*opt*_ features that were selected most often when creating a feature set of size *n*_*opt*_ by RFECV. The features selected for each model are listed in Table 3.

**Table 3:** Optimal feature sets identified by repeating recursive feature elimination with cross‐validation.^a^

	Models						
Features^b^	L0	L1	L2	L3	L4	Scale space	Count
Green REFL SD						L0 L1 L2 L3	1
Green ASM mean	✓	✓				L0 L1 L2	3
Green ASM range		✓				L0 L1 L2	2
Green COR range						L3	1
Green DENT mean						L0 L1 L2	1
Green ENT mean	✓	✓	✓			L0 L1 L2	4
Green SENT mean	✓					L0 L1 L2	2
Red REFL SD						L0	1
Red ASM mean		✓				L1 L2	2
Red COR range						L3	1
Red DENT range						L0 L1	1
Red DENT range						L2	1
Red ENT mean						L0 L1	1
Red SENT mean						L0	1
Red SENT range						L4	1
Red‐edge REFL mean	✓	✓				L0 L1 L2 L3 L4	3
Red‐edge ASM mean						L2 L4	1
Red‐edge COR range						L1 L2	1
Red‐edge DENT range						L2	1
Red‐edge ENT mean						L2 L4	1
Red‐edge ENT range						L1	1
NIR REFL mean	✓	✓	✓	✓	✓	L0 L1 L2 L3 L4	6
NIR ASM range						L0	1
NIR COR mean						L4	1
NIR ENT range						L1	1
NIR SENT range						L0 L1 L3	1
NDVI ASM mean						L1 L2	1
NDVI ASM range						L1	1
NDVI DENT mean						L2 L3	1
NDVI ENT mean		✓	✓			L1 L2	3
NDVI ENT range						L1	1
NDVI SENT mean						L2	1
NDVI SENT range						L0 L3	1
gNDVI ASM mean						L3	1
gNDVI ASM range						L3	1
gNDVI DENT range						L2 L3	1
gNDVI ENT mean						L2 L3 L4	1
gNDVI ENT range						L2	1
gNDVI SENT range	✓					L0	2
reNDVI VAL mean		✓				L0 L1 L2 L3 L4	2
reNDVI COR mean		✓				L1 L2 L3 L4	2
reNDVI COR range						L3	1
reNDVI DENT mean						L2	1
reNDVI DENT range						L2	1
reNDVI ENT range						L3	1
reNDVI SENT range						L3	1

^a^A check mark in the L0, L1, L2, L3, or L4 column indicates that the feature was selected for use in that model. The code(s) L0–L4 in the scale‐space column indicate the level(s) of aggregation of the feature selected for use in the scale‐space model. Finally, the count column indicates the number of different models (L1–L4) and scale space in which a feature was selected for use.

^b^ The features are named using the raster variable names: green, red, red‐edge, near infrared (NIR), normalized difference vegetation index (NDVI), green NDVI (gNDVI), and red‐edge NDVI (reNDVI), combined with the variable names: angular second moment (ASM), correlation (COR), difference entropy (DENT), entropy (ENT), sum entropy (SENT), vegetation index value (VAL), and reflectance (REFL).

### Hyperparameter optimization

Following feature optimization, a grid search was used to identify the optimal hyperparameters for developing a model using these feature sets. The tuned values for these hyperparameters are listed in Appendix S6. The optimal values selected for the hyperparameters fell within the range of values explored by the grid search, as opposed to at an endpoint, suggesting that the values identified will fall close to the optimal region within the hyperparameter space.

Following hyperparameter tuning, the models were tested using the 31 examples in the independent validation dataset. The accuracy of the models on the training and validation sets is listed in Table 4. The confusion matrix resulting from applying the L0 and scale‐space classifiers (the best single‐level and best overall classifiers) to the validation data is shown in Table 5. Because of the imbalance in the validation data class membership, the overall accuracy (see Table 4) was dominated by the model's performance on the High and Moderate classes and was not a robust indicator of the model's performance on the Low class. Using the data from Table 5, the macro‐averaged F1 score, which is commonly used to measure performance on imbalanced datasets (Farhadpour et al., 2024), was calculated to be 0.60 and 0.83 for the L0 and state‐space models, respectively. This result indicates that the state‐space model performed much better than the L0 model.

**Table 4:** The overall accuracy of the single‐scale and scale‐space models on the training and independent validation sets.

Model level	Training	Validation
0	0.513	0.677
1	0.435	0.645
2	0.469	0.484
3	0.332	0.355
4	0.303	0.419
Scale space	0.560	0.742

**Table 5:** Confusion matrix of the classification of independent validation data by the tuned L0 and scale‐space models.

			Predicted class		
Model			Low	Moderate	High
L0	True Class	Low	1	0	0
		Moderate	2	5	6
		High	0	2	15
Scale space	True Class	Low	1	0	0
		Moderate	0	7	6
		High	0	2	15

Finally, classification maps derived from the results of applying the optimized L0 and scale‐space classifiers to the orthomosaicked image from field site 3 on 4 July are illustrated in Figure 6. The differences in these classification maps are due to the different features available to the L0 classifier compared to the state‐space classifier. The L0 classifier could only leverage features derived from images with a spatial resolution of 2.9 cm, whereas the state‐space classifier could leverage features derived from all five spatial resolutions in the image GPs.

**Figure 6:**
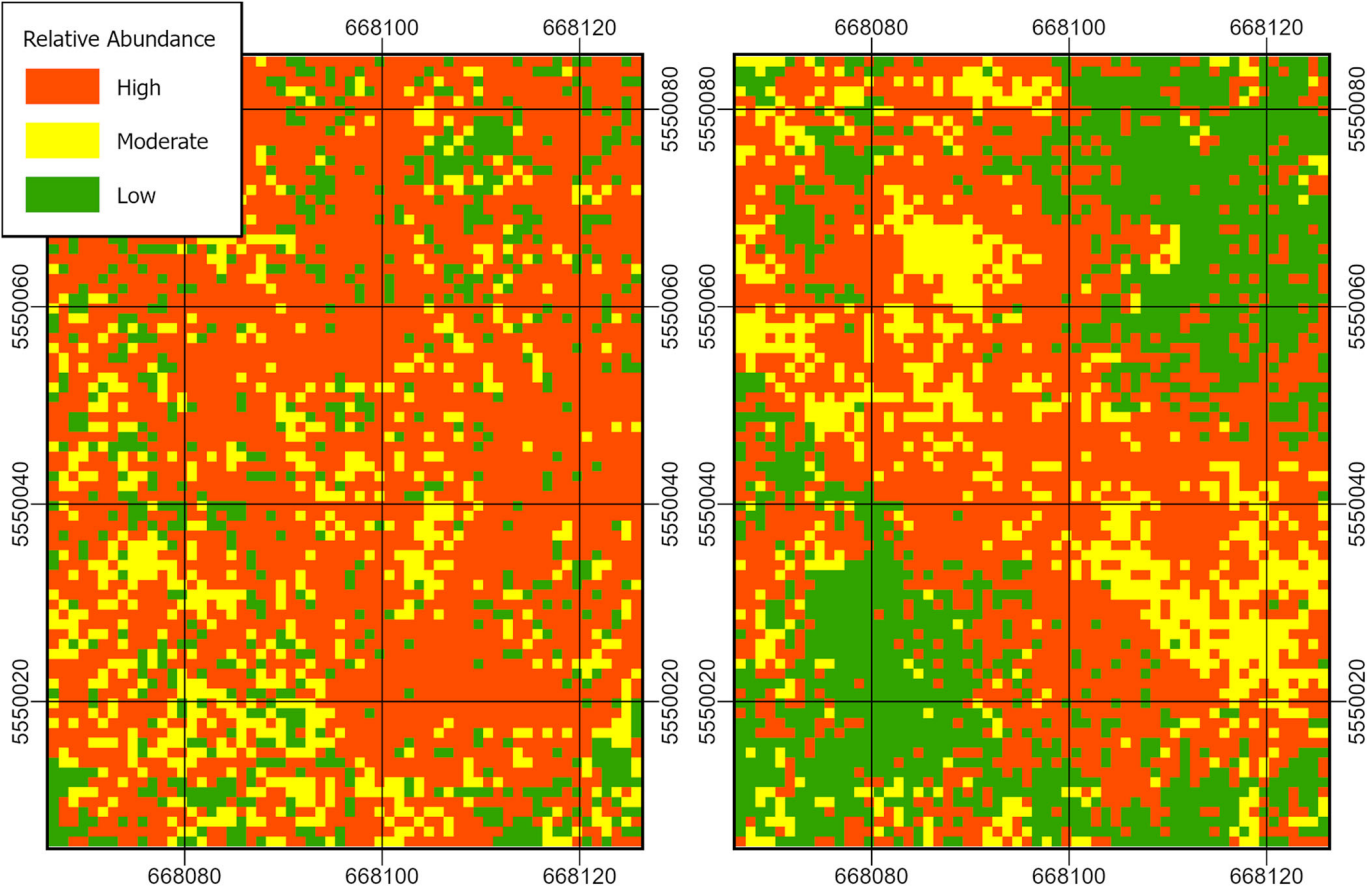
The classification map generated by the single‐scale L0 model (left) and the scale‐space model (right). The features used to classify the 1‐m^2^ metapixels in the L0 model have a 2.9‐cm resolution, whereas features driving the scale‐space model have spatial resolutions ranging from 2.9 cm to 46 cm as listed in Table 1. Images are referenced to the WGS UTM Zone 10N coordinate system.

## DISCUSSION

The objective of this study was to determine whether there is a relationship between spatial resolution and image classification accuracy that can help identify an optimal spatial resolution for mapping spotted knapweed using multispectral imagery and to determine whether providing the image features at multiple spatial resolutions further improves this classification. The classifier performance measures (see Table 4 **)** reveal that the best single‐scale classifier was constructed using the highest spatial resolution (L0) feature set and that the classification performance increasingly degrades as the spatial resolution is decreased. This result suggests that remotely sensed imagery should be acquired at the highest spatial resolution possible given financial, labor, and logistical constraints. However, these results also show that the best classifier was not produced using only the highest spatial resolution data, but rather by the scale‐space classifier, which leveraged features revealed by image data with multiple spatial resolutions. By using image GPs to simulate these multi‐resolution image datasets, rather than physically collecting them, the state‐space classifier presented here can be used without increasing the existing financial, labor, and logistical costs of RPAS‐based remote sensing.

The use of multi‐scale features derived from image GPs increased the performance of the state‐space classifier on the validation dataset by 6.5% over the single‐resolution L0 classifier. The confusion matrices of these two classifiers reveal that the performance gain is entirely due to an increase in the ability to classify the Moderate relative abundance category (Table 5). It can be argued that this category is more difficult to identify in the spectral imagery than either the Low or High categories, because at moderate relative abundances, the location and configuration of spotted knapweed will exhibit more spatial heterogeneity within a 1‐m^2^ metapixel than it can in a metapixel with a low or high relative abundance.

The improvement in classification accuracy has a dramatic effect on the map of the spotted knapweed infestation of site 3 on 4 July created using the scale‐space classifier, compared to the L0 classifier (Figure 6). The L0 model replicates the model published by Baron and Hill (2020) and creates a map of spotted knapweed relative abundance that indicates the site is predominantly heavily infested, with some areas of moderate abundance clustered towards the southwest corner of the site and with a few regions of low abundance scattered throughout the site. The speckled appearance of the L0 map suggests that the relative abundance of spotted knapweed is not spatially correlated, but is instead randomly distributed through the site. However, this is not a realistic spatial model of the relative abundance of the plant, which tends to grow in clusters. The scale‐space model, on the other hand, produces a map of the relative abundance of spotted knapweed within site 3 that exhibits considerable spatial structure of the regions of relative abundance. The scale‐space model predicts two primary regions of low abundance in the southwest and northeast corners of the site, with a central band of high abundance running through the center of the site. This map also has significantly less speckle than the map produced using the L0 model, resulting in a more natural looking distribution of spotted knapweed throughout the region. Furthermore, the regions of relative abundance predicted by the scale‐space model align with the spatial structure of the vegetation canopy visible within the aerial photo of site 3 taken on 4 July 2018 (see Appendix S5).

In addition to creating a superior classification model, the scale‐space modeling process also provides insights to the spatial scale dependency of important features for classifying spotted knapweed. Of the 84 single‐scale features, no more than nine (11%) are selected for use in at least one single‐scale classification model, while only 89 (21%) of the 420 scale‐space features are selected for the scale‐space classification model (Table 2). This suggests that there are many irrelevant or redundant features present in the feature sets. Furthermore, the 89 scale‐space features selected constitute only 46 different statistics of the base spectral reflectance or vegetation index data. These results suggest that the utility of these features for classifying spotted knapweed is dependent on the spatial scale at which these features are provided for random forest model training.

The relevant spatial scales for features can be identified in Table 3 by looking at the features selected for each of the single‐scale models. Some features are relevant only at specific spatial scales, namely: green SENT, gNDVI SENT, green ASM mean, green ASM range, green ENT mean, red ASM mean, NDVI ENT mean, reNDVI value mean, and reNDVI COR mean. Green SENT mean and gNDVI SENT range are only valuable at the highest spatial resolution (L0). The SENT statistic quantifies the complexity of the spatial organization of high‐ and low‐valued pixels of the variable being summarized, in this case, the green band reflectance and the gNDVI. This result is likely caused by variability in the green and NIR spectral bands being smoothed out as the pixel size increases within the image scale spaces. While not relevant at the base resolution (i.e., L0), the variables green ASM mean, green ASM range, green ENT mean, red ASM mean, NDVI ENT mean, reNDVI value mean, and reNDVI COR mean emerge as predictors at the L1 spatial scale. Moving to higher levels within an image GP not only decreases the spatial resolution of the data used to calculate the GLCM‐derived features but also increases the spatial footprint of the kernel over which the feature is computed. Thus, GLCM‐derived statistics computed at higher levels within the GP capture patterns that persist over longer length scales than the same statistics computed at lower levels within the GP. The importance of these statistics related to homogeneity (ASM and ENT) and linear relationships between neighboring pixels (COR) at higher levels of the GP but not at the base (L0) level suggests that the 1‐pixel neighborhood of the GLCM is too small at the L0 spatial resolution (2.9 cm) and that these features do not become sufficiently descriptive until spatial scales of approximately 6 cm.

Features including green ASM mean, green ENT mean, red‐edge mean reflectance, NIR mean reflectance, and NDVI ENT mean are selected over neighboring levels within the image GPs, suggesting that these features persist over finite regions of the feature scale spaces. Of these features that remain important for classifiers built using features computed at multiple levels within the GP, NIR mean reflectance and green ENT mean are of note. The NIR mean reflectance is the only feature selected for use in all five of the single‐scale models. This suggests that NIR mean reflectance is the most important predictor of the relative abundance of spotted knapweed (in fact, the L3 and L4 models use only this feature); moreover, it indicates that this variable is a robust predictor across all of the spatial scales explored in this analysis. The green band mean ENT is the second most common feature, appearing in three of the five models (L0, L1, and L2). This result also suggests that these two features should be prioritized when designing RPAS‐based data collection protocols for mapping spotted knapweed. Furthermore, the common use of features derived from the NIR and green spectral bands suggests that the chlorophyll absorption and moisture content of the vegetation, two characteristics related to the NIR and green band reflectance, are important indicators of the relative abundance of spotted knapweed in our study site.

The utility of NIR and green spectral bands for predicting the relative abundance of spotted knapweed may be because this exogenous plant behaves differently, and thus will have a different spectral signature, during the hot dry summers of the BC interior than the native vegetation. Native vegetation present in the study site have evolved to slow down photosynthesis to conserve water during the summer, whereas spotted knapweed is known to actively photosynthesize during this time, so it is expected that in these images native vegetation will reflect more NIR and less green than spotted knapweed. The importance of green band ENT indicates that the complexity of the spatial pattern of green reflectance is a significant feature for classifying spotted knapweed relative abundance. This result could be related to the structure of spotted knapweed, which grows tall shoots with dark green leaves that overhang underlying ground cover, a situation that would result in high variation in green reflectance between neighboring pixels depending on whether the pixel represents spotted knapweed or the underlying vegetation. Surprisingly, mean red reflectance, typically a vital component in traditional vegetation indices like NDVI, is absent from the optimized feature sets. This omission might indicate that the models leverage other features that capture similar information. For example, the reNDVI, which is formulated using the NIR and RE bands, appears in the L0 and scale‐space feature sets.

An analysis of the features selected for use in the scale‐space model reveals similar patterns as revealed by the single‐scale models. Of the features that were included in the optimal feature set of the scale‐space model, half (23) were selected at more than one spatial scale, while the other half were selected at only one spatial scale. Furthermore, every feature selected by a single‐scale model was selected by the scale‐space model at the same scale. However, the scale‐space model also selects features at multiple scales that were not picked by more than one of the single‐scale models. As already discussed, features that are selected at many spatial scales are less scale‐dependent than features that are selected at only one spatial scale. However, the frequency with which the scale‐space model selects features at multiple spatial scales suggests that multicollinearity between the features at different spatial scales can lead to bloating of the optimal feature set when using random forest–based RFE. This is likely the result of the random forest algorithm using a randomized subset of features to grow each tree in its forest, where the number of features in the subset is equal to the square root of the total number of features available to the random forest algorithm (Probst et al., 2019). Because the trees in the random forest are not allowed to use either the full set of features or the same subset of features, it is expected that they will identify different features as important. However, if an important feature is redundant (i.e., a scale‐independent feature computed at multiple spatial resolutions), this feature will be identified as important at whatever scale(s) it is provided, and thus the random forest algorithm will rank this feature as being important at many spatial scales.

This analysis of the optimized feature sets reveals that feature importance is dependent on feature spatial resolution. Some features are only useful at high resolutions, whereas others are only useful at lower resolutions. This contradicts the common assumption that higher spatial resolution data will always improve analytical performance and instead suggests that the spatial resolution of features must be optimized to produce the best remote sensing classifier. This is particularly important in the case of GLCM‐derived features, as the spatial scale of these features is related to the length scale of the pattern quantified by the statistic. This result has important implications for selecting flight heights for data collection. Flights conducted to produce image data with resolutions higher than the critical length scale of these features must be aggregated to extract these features, while flights conducted to produce image data with resolutions lower than the critical length scale of these features cannot leverage these features for classification. This result reinforces the practice of planning imaging missions to collect the highest spatial resolution imagery feasible given cost and time constraints. However, scale‐space analysis could be performed using data collected at the plot scale to identify the optimal image spatial resolution(s), which could be used to calculate (based on the RPAS specifications) the maximum flight height allowable for a given image acquisition mission over a broader region.

Finally, this analysis reveals that spotted knapweed monitoring and management could be enhanced by using maps produced by classifiers leveraging scale‐space representation of the RPAS‐acquired multispectral imagery. Combining flight height optimization with plot‐scale ground‐reference data collection and classifier training will improve efficiency of RPAS imaging missions over the management area. By using image GPs to simulate multi‐scale image collection, scale‐space models can be constructed without additional resources for data collection in the field. While the present study demonstrates the utility of scale‐space models for classifying spotted knapweed within grasslands occurring in BC's IDFxh2 biogeoclimactic zone, we expect that the methods presented here, which employ GP‐derived multi‐scale features to drive metapixel‐based image analysis, will be useful for spotted knapweed mapping in grasslands occurring in other biogeoclimactic conditions. Furthermore, we expect that these methods may also be useful for identifying other vegetative invasive species, such as cheatgrass (*Bromus tectorum* L.), in grassland ecosystems. In either of these potential extensions of this work, the statistics listed in Appendix S4 will provide a good initial set of features to be optimized, which will result in an optimal multi‐scale feature set customized to the new target species and/or biogeoclimactic conditions.

## AUTHOR CONTRIBUTIONS

S.S. and D.J.H. developed the study concept and methodology. Data preparation and analysis was performed by S.S. with input from D.J.H. Validation of the results and conclusions was performed by D.J.H. The first draft of the manuscript was prepared by S.S. with review and editing by D.J.H. Both authors approved the final version of the manuscript.

## Supporting information


**Appendix S1:** Remotely piloted aircraft system (RPAS) flight parameters used for data collection.
**Appendix S2:** Definitions of the parameters of the random forest classifier and the values within the parameter space explored by grid search during hyperparameter tuning.
**Appendix S3:** Gray‐scale collocation matrix statistics.
**Appendix S4:** Features describing each image metapixel. Each of these values is calculated for each of the seven rasters.
**Appendix S5:** Aerial image of field site 3 generated from imagery collected on 4 July 2018 by the onboard digital camera.
**Appendix S6:** Values of tuned random forest hyperparameters for the single‐scale and scale‐space models.

## Data Availability

Data are available for use under the CC‐BY Attribution 4.0 International license and can be downloaded from the Open Science Framework (OSF) at https://doi.org/10.17605/OSF.IO/M2A4D.
