# Supplementary material for: Scale‐space image analysis for mapping spotted knapweed in grassland ecosystems
Source: Appl Plant Sci. 2026 Oct 14:e70086. Online ahead of print. doi: 10.1002/aps3.70086 (PMC13669681; doi:10.1002/aps3.70086)
Supplement: Supplementary file 1 [file APS3-9999-e70086-s001.docx]

# **Appendix S1.** Remotely piloted aircraft system (RPAS) flight parameters used for data collection.

| **Height above**  **ground level (m)** | **Forward overlap (%)** | **Side overlap**  **(%)** | **Time of day** | **Max. wind speed (km/h)** |
| --- | --- | --- | --- | --- |
| 30 | 80 | 80 | 11:00 am to  1:00 pm | 40 |

# **Appendix S2.** Definitions of the parameters of the random forest classifier and the values within the parameter space explored by grid search during hyperparameter tuning.

| **Parameter** | **Definition** | **Values explored** |
| --- | --- | --- |
| n_estimators | Number of trees in the forest | 500, 1000, 1500, 2000, 2500 |
| max_depth | Maximum depth of the tree | 20, unlimited |
| min_samples_split | Minimum number of samples required to split an internal node | 2, 3 |
| min_samples_leaf | Minimum number of samples required to be at a leaf node | 1, 2, 3 |

# **Appendix S3.** Gray-scale collocation matrix statistics.

Distribution of gray levels along the horizontal axis:

$$p_{x}\left( i \right)= \sum_{j=1}^{N_{g}} p\left( i, j \right)$$

(S3.1)

where $i$ is a horizontal position in the image. $j$ is a particular gray-level intensity value. *N_g_* is the number of possible gray-level intensities. $p\left( i, j \right)$ represents the probability of finding a pixel with gray level $j$ at the horizontal position $i$. $p_{x}\left( i \right)$ represents an average gray-level intensity at horizontal position $i$, considering all vertical positions in the image.

The mean of gray levels along the horizontal axis:

$$\mu_{x}= \frac{1}{N_{g}}\sum_{i=1}^{N_{g}} p_{x}\left( i \right)$$

(S3.2)

Standard deviation of gray levels along the horizontal axis:

$\sigma_{x}=\sqrt{\frac{1}{N_{g}-1}\sum_{i=1}^{N_{g}} \left( p_{x}\left( i \right)-\mu_{x} \right)^{2}}$

(S3.3)

The distribution of gray levels along the vertical axis:

$$p_{y}\left( j \right)= \sum_{i=1}^{N_{g}} p\left( i, j \right)$$

(S3.4)

where $p_{y}\left( j \right)$ represents an average gray-level intensity at vertical position $j$, considering all horizontal positions in the image.

The mean of gray levels across the vertical axis:

$$\mu_{y}= \frac{1}{N_{g}}\sum_{j=1}^{N_{g}} p_{y}\left( j \right)$$

(S3.5)

The standard deviation of gray levels across the vertical axis:

$$\sigma_{y}= \sqrt{\frac{1}{N_{g}-1}\sum_{i=1}^{N_{g}} \left( p_{y}\left( i \right)-\mu_{y} \right)^{2}}$$

(S3.6)

Because of the assumption of symmetry in the angular relationships considered in this work, $\mu_{x}=\mu_{y}$ and $\sigma_{x}=\sigma_{y}$.

The sum of the distribution gray levels:

$$\begin{matrix} p_{x+y}\left( k \right)= \sum_{i=1}^{N_{g}} \sum_{j=1}^{N_{g}} p\left( i, j \right) \\ k=i+j k=2,3,\ldots,2N_{g} \end{matrix}$$

(S3.7)

where $p_{x+y}\left( k \right)$ represents the probability distribution of the sum of gray-level intensities of pixels at positions $i \mathrm{and} j$, and signifies the likelihood of encountering a specific sum of gray levels in the image.

The distribution of gray-level differences:

$$\begin{matrix} p_{x-y}\left( k \right)= \sum_{i=1}^{N_{g}} \sum_{j=1}^{N_{g}} p\left( i, j \right); \\ k=\left| i-j \right| k=0,1,\ldots,N_{g}-1 \end{matrix}$$

(S3.8)

where $p_{x-y}\left( k \right)$ represents the probability distribution of a specific difference in gray-level intensities of pixels at position $i \mathrm{and} j$.

From the values calculated in equations 6–13, the second-order statistics of ASM, COR, ENT, SENT, and DENT can be calculated:

$ASM= \sum_{i=1}^{N_{g}} \sum_{j=1}^{N_{g}} \left\{ p\left( i, j \right) \right\}^{2}$

(S3.9)

where $p\left( i, j \right)$ represents the probability (or normalized frequency) that a pixel with gray level $i$ is adjacent to a pixel with gray level $j$ in a particular direction (e.g., horizontal, vertical, diagonal). Squaring the probabilities emphasizes higher probabilities, which, in the context of the ASM, gives a measure of uniformity.

$$COR=\frac{\sum_{i=1}^{N_{g}} \sum_{j=1}^{N_{g}} \left( i* j \right)p\left( i, j \right)-\mu_{x}\mu_{y}}{\sigma_{x}\sigma_{y}}$$

(S3.10)

$$ENT= -\sum_{i=1}^{N_{g}} \sum_{j=1}^{N_{g}} p\left( i, j \right){log}_{2}\left[ p\left( i, j \right)+\varepsilon\right]$$

(S3.11)

where $\varepsilon$ is a small constant value to ensure that the logarithm is defined (preventing log of zero, which is undefined), which ensures stability in the computation, especially for cases where $p\left( i, j \right)$ might be zero.

$$SENT= -\sum_{i=2}^{{2N}_{g}} p_{x+y}\left( i \right){log}_{2}\left[ p_{x+y}\left( i \right)+\varepsilon\right]$$

(S3.12)

where $\varepsilon$ is a small constant value to ensure that the logarithm is defined (preventing log of zero, which is undefined), which ensures stability in the computation, especially for cases where $p\left( i, j \right)$ might be zero.

$$DENT= -\sum_{i=0}^{N_{g}-1} p_{x-y}\left( i \right){log}_{2}\left[ p_{x-y}\left( i \right)+\varepsilon\right]$$

(S3.13)

where $\varepsilon$ is a small constant value to ensure that the logarithm is defined (preventing log of zero, which is undefined), which ensures stability in the computation, especially for cases where $p\left( i, j \right)$ might be zero.

# **Appendix S4.** Features describing each image metapixel. Each of these values is calculated for each of the seven rasters.

| **Name** | **Definition** |
| --- | --- |
| Mean | Mean of values |
| SD | Standard deviation of values |
| meanASM | Mean GLCM angular second moment |
| rangeASM | Range GLCM angular second moment |
| meanCOR | Mean GLCM correlation |
| rangeCOR | Range GLCM correlation |
| meanENT | Mean GLCM entropy |
| rangeENT | Range GLCM entropy |
| meanSENT | Mean GLCM sum entropy |
| rangeSENT | Range GLCM sum entropy |
| meanDENT | Mean GLCM difference entropy |
| rangeDENT | Range GLCM difference entropy |

# **Appendix S5.** Aerial image of field site 3 generated from imagery collected on 4 July 2018 by the onboard digital camera.

# **Appendix S6.** Values of tuned random forest hyperparameters for the single-scale and scale-space models.

|  | Model | | | | | |
| --- | --- | --- | --- | --- | --- | --- |
| Parameter | L0 | L1 | L2 | L3 | L4 | Scale-Space |
| n_estimators | 1000 | 2000 | 2000 | 500 | 500 | 2000 |
| max_depth | 20 | 20 | 20 | 20 | 20 | 20 |
| min_samples_split | 3 | 2 | 2 | 2 | 2 | 2 |
| min_samples_leaf | 1 | 3 | 2 | 3 | 2 | 1 |
